# Leptin and interleukin-1β levels associated with osteoarthritis in Vietnamese patients: a cross-sectional analysis

**DOI:** 10.1590/1414-431X2023e12746

**Published:** 2023-09-08

**Authors:** N.T.T. Mai, N.T. Hang, D.H. Hanh, H.Y. Le, N.V. Hinh, N.D. Ky, N.M. Tuan, H.V. Tong, D.T. Quan, N.L. Toan

**Affiliations:** 1 Bach Mai Hospital, Hanoi, Vietnam; 2 Department of Pathophysiology, Vietnam Military Medical University, Hanoi, Vietnam; 3 108 Military Central Hospital, Hanoi, Vietnam; 4 Department of Endocrinology, Nghe An Friendship General Hospital, Nghe An, Vietnam; 5 National Hospital of Endocrinology, Hanoi, Vietnam; 6 Institute of Biomedicine and Pharmacy, Vietnam Military Medical University, Hanoi, Vietnam

**Keywords:** Osteoarthritis, Metabolic syndrome, Leptin, Interleukin-1β, HbA1c

## Abstract

Leptin and interleukin-1 beta (IL-1β) are two extensively studied biomarkers associated with metabolic syndrome (MetS) and osteoarthritis (OA). Previous studies have mostly focused on either MetS or OA alone, with no available data on Vietnamese patients. This study aimed to investigate the levels of leptin and IL-1β in this patient population and explore their association with clinical parameters of MetS and OA. The study included 164 patients with primary knee OA, who were classified into two categories based on the presence of MetS, and 78 healthy controls. The plasma leptin and IL-1β levels were quantified by ELISA and correlated with clinical parameters. Leptin levels were higher in patients with OA (11.50±10.04 ng/mL) than in healthy controls (0.54±0.37 ng/mL) and increased in patients with MetS compared to those without MetS. IL-1β levels were also significantly higher in OA patients (14.63±15.87 pg/mL) than in controls (7.79±5.11 pg/mL), but were not significantly different between the MetS and non-MetS groups. Leptin levels were positively correlated with body mass index, waist-to-hip ratio, visual analogue scale scores, HbA1c and insulin levels, and HOMA-IR index, whereas IL-1β levels were only correlated with insulin levels and HOMA-IR index. ROC curve analysis revealed that leptin and IL-1β levels could distinguish individuals with and without OA (AUC=0.96; 0.88, respectively), and individuals with and without MetS (AUC=0.82; 0.71, respectively). Our findings suggested that both leptin and IL-1β levels were associated with both MetS and OA and may play a critical role in the pathogenesis of MetS-related OA.

## Introduction

Osteoarthritis (OA) is a degenerative joint disease with a high prevalence in the United States and worldwide and is recognized as a leading cause of functional disability (1). OA primarily affects adults aged 65 and older and the knee is the most commonly affected joint (2). According to the National Health Interview Survey, approximately 13.7 million individuals in the USA had symptomatic knee OA in 2007-2008, and this number increased to 15.1 million in 2011-2012 (3). In Vietnam, although the prevalence of OA has not been reported, a study with a large sample size in urban areas indicated that 18.2% of people aged 16 and older experienced knee pain (4). Furthermore, another study found that the prevalence of radiographic OA of the knee was 34.2% among 658 participants (5). Due to the aging of the population and the obesity epidemic, the number of people affected by symptomatic knee OA is likely to increase (2). This could pose a challenging problem for the healthcare system to deal with this disease in Vietnam and worldwide.

In a previous study, the group with a higher body mass index (BMI) had a higher risk of knee OA, with up to two-thirds of the elderly obese population being affected by knee OA (6). This can be explained by the increased joint loading and changes in body composition that have detrimental effects related to meta-inflammation and behavioral factors, such as diminished physical activity and subsequent loss of protective muscle strength (7). Studies have shown that metabolic syndrome (MetS) and its components, including obesity, hypertension, and hyperglycemia, are associated with knee OA (8-10). A recent review has also concluded that MetS has been associated with OA (11). Although the exact mechanism of this correlation is not yet clear, studies suggest that MetS influences the pathogenesis of OA through a wide range of metabolic alterations that directly affect macrophages and chondrocytes (11,12). MetS has been reported to be associated with an increased risk of developing OA (13) and with increased levels of leptin and interleukin-1 beta (IL-1β) in the plasma (14), suggesting that the presence of MetS may promote the development and progression of OA. Additionally, race has been found to be related to the prevalence of MetS among patients with knee OA (15).

In obesity, adipose tissue secretes various adipokines, including leptin and adiponectin, as well as pro-inflammatory cytokines (16). Leptin plays a crucial role in energy metabolism by regulating appetite and increasing energy consumption (17). Since leptin is predominantly secreted by adipocytes, its level is largely influenced by the white adipose tissue mass and BMI (18,19). As a result, leptin levels are frequently elevated in obese individuals. Moreover, leptin resistance results in negative feedback on leptin concentration, leading to a continuous reduction in leptin sensitivity and an increase in leptin secretion (17). In addition, the literature suggests that the serum leptin level is positively correlated with OA, and partially mediates the association between adiposity and OA (17,20). Furthermore, both high serum and synovial fluid leptin levels are associated with OA symptoms, such as joint pain and loss of physical function, and radiographic severity of OA (21-24).

Pro-inflammatory cytokines, including tumor necrosis factor-alpha (TNF-α), IL-1β, and IL-6, are mainly produced by macrophages in fat tissue (25). Abnormally elevated levels of these cytokines have been observed in the synovial membrane, synovial fluid, subchondral bone, and cartilage of OA patients, indicating their vital role in the pathogenesis of OA (16). These cytokines can promote the production of other cytokines, prostaglandins, and matrix metalloproteinases, while inhibiting the synthesis of proteoglycans and type II collagen. Therefore, they play an important role in cartilage matrix degradation and bone resorption in OA. Additionally, these cytokines can indirectly cause OA by regulating the release of adipokines from adipocytes (16).

Although leptin and IL-1β have been extensively studied as potential markers, most studies have focused on patients with either MetS or OA alone. However, the role of these markers in relation to the clinical outcomes of both MetS and OA needs to be validated in different populations. Furthermore, no study to date has reported on leptin and IL-1β levels in Vietnamese patients with primary OA and MetS. Therefore, the present study aimed to investigate the plasma levels of leptin and IL-1β, as well as their correlations with clinical parameters, in Vietnamese patients with primary knee OA and MetS.

## Material and Methods

### Study population

A total of 164 patients with primary knee OA and 78 control individuals were enrolled at Bach Mai Hospital in Hanoi, Vietnam from January 2014 to December 2019. Primary knee OA was diagnosed according to the clinical and radiographic criteria of the American College of Rheumatology. The patients were diagnosed with primary knee OA if they had joint symptoms such as pain, aching, or stiffness in a joint and the presence of at least one of the radiographic features in the affected joint such as osteophytes, joint space narrowing, subchondral sclerosis, and cysts (26). The patients were classified into two subgroups based on MetS status: OA with MetS (n=85) and OA without MetS (n=78). MetS was defined as the presence of at least three of the following risk factors: increased blood pressure, increased fasting glucose, dyslipidemia (increased triglycerides and reduced HDL), and central obesity (27).

Patients with secondary OA, liver cirrhosis, kidney and heart failure, infectious diseases, and urgent conditions, such as paroxysmal hypertension, acute brain stroke, gastrointestinal bleeding, or more severe complications, were excluded. Patients who were taking any medication, which might affect biochemical results, such as glucocorticoid, diacerein, statins, thiazide diuretics, angiotensin II receptor blockers, and angiotensin-converting enzyme inhibitors, had to temporarily stop receiving medical treatment for at least a week prior to sampling. In addition, we excluded patients who were taking analgesic and/or non-steroidal anti-inflammatory drugs. For the control group, individuals had to be healthy with blood pressure <135/85 mmHg, fasting venous blood glucose <5.6 mmol/L, and BMI ranging from 18.5-25. None of them had abnormal results of biochemical tests, any chronic liver, lung, kidney, or heart diseases, infectious conditions, or MetS.

All participants underwent routine clinical examinations and biochemical tests. We recorded the clinical characteristics of all enrolled patients, such as blood pressure, BMI, and waist-to-hip ratio (WHR). In addition, the number of involved joints, disease duration, visual analogue scale (VAS), and the Western Ontario and McMaster Universities Arthritis Index (WOMAC) were obtained by using the standard study questionnaires (28). Disease duration of fewer than 60 months was defined as “short” and ≥60 months was defined as “long”. The number of affected joints was evaluated by physical examination, radiographic imaging, and questionnaire.

Informed written consent was received from all participants. The study was approved by the Institutional Review Board of the Vietnam Military Medical University (VMMU).

### Measurement of biochemical parameters

The measurement of lipid components levels, including triglycerides, total cholesterol, high-density lipoprotein-cholesterol (HDL-C), and low-density lipoprotein-cholesterol (LDL-C) was performed using an automatic biochemical Cobas 6000 Chemistry Analyzer Series (Roche Diagnostics GmbH, Germany). Insulin levels were measured using the Cobas 8000 Modular Analyzer Series (Roche Diagnostics GmbH) based on the electrochemical immune fluorescence method. The concentrations of fasting blood glucose were quantified by ultraviolet measurement with hexokinase. The blood fasting glycosylated hemoglobin (HbA1c) levels were measured by the Tosoh Automated Glycohemoglobin Analyzer HLC-723G8 (Tosoh, Japan) through the ion-exchange method using high-performance liquid chromatography. The homeostasis model assessment insulin resistance (HOMA-IR) index was calculated according to Matthews' method: HOMA-IR = glucose × insulin / 22.5 (29).

### Measurement of leptin and IL-1β levels

Leptin levels were quantified in the respective plasma samples of enrolled patients using a commercial enzyme-linked immunosorbent assay (ELISA) kit according to the manufacturer's instruction (Human Leptin, Catalog Number: RAB0333; Sigma-Aldrich, USA). IL-1β levels were quantified in the respective plasma samples of enrolled patients using commercially available ELISA kits according to the manufacturer's instruction (Human IL-1, Catalog Number: DLB50; Bio-Techne China Co., Ltd., China).

### Statistical analysis

Continuous variables are reported as median values and range, and categorical variables are reported as numbers and percentages. The clinical and demographic characteristics between groups were compared using *t*-test and one-way ANOVA for continuous variables and *χ* ^2^ test and Fisher's exact test for categorical variables. The plasma levels of leptin and IL-β were analyzed between OA patients and controls by the Kruskal-Wallis and Mann-Whitney U tests. Spearman's rank correlation coefficient was used to analyze the correlation between two variables as well as between markers and clinical parameters. Receiver operating characteristic (ROC) analysis and the area under the curve (AUC) calculation were performed to assess whether leptin and IL-1β levels could distinguish individuals with and without OA and individuals with and without MetS. The statistical significance was set at a P-value of less than 0.05. All analyses were performed with SPSS statistical software version 25.0 (IBM, USA).

## Results

### Baseline characteristics

The baseline characteristics of the patients with OA and controls are shown in Table 1. The mean age was higher for OA patients (57.7±8.1 years) than controls (37.2±9.8 years, P<0.001), and the mean age for MetS OA patients was higher than non-MetS OA patients (P=0.014). The incidences of female and bilateral knee OA patients were not different between groups as well as between subgroups. The levels of triglycerides, total cholesterol, and LDL-C were significantly higher in the OA patients compared with controls (P<0.001), whereas the levels of fasting glucose and HDL-C were not different between groups (P>0.05). Blood pressure, BMI, waist/hip ratio (WHR), VAS, and WOMAC were significantly different between OA patients and controls (P<0.05). The baseline characteristics of the non-MetS-OA group were also compared to those of the control group. The results of this comparison showed a similar pattern to that observed in the comparison between all OA patients and controls (Table 1).

**Table 1:** Characteristics of patients with osteoarthritis (OA), with and without metabolic syndrome (MetS), and controls.

Characteristics	Osteoarthritis			Control(n=78)	P value**	P value***
	MetS OA (n=85)	Non-MetS OA (n=79)	P value*			
Age (years)	60 (40-72)	56 (38-74)	0.014	36 (19-58)	<0.001	<0.001
Female sex	77 (90.6)	64 (81.0)	NS	67 (85.9)	NS	NS
Bilateral knee OA	60 (70.6)	50 (63.3)	NS	NA	NA	NA
Disease duration (months)	24 (1-240)	12 (1-240)	0.025	NA	NA	NA
SBP (mmHg)	140 (110-220)	120 (90-210)	<0.001	110 (90-125)	<0.001	<0.001
DBP (mmHg)	90 (60-130)	80 (60-130)	0.002	70 (60-80)	<0.001	<0.001
BMI	25.1 (20.8-35.7)	22.7 (16.3-31.2)	<0.001	20.8 (18.1-23.4)	<0.001	<0.001
WHR	0.96 (0.87-1.15)	0.90 (0.74-1.14)	<0.001	0.83 (0.69-0.96)	<0.001	<0.001
VAS	6 (2-9)	4 (1-9)	0.006	NA	NA	NA
WOMAC	25 (4-67)	20 (3-62)	0.016	NA	NA	NA
Fasting glucose (mmol/L)	5.8 (4.1-12.1)	5.3 (4.4-11.1)	0.021	5.2 (4.3-5.9)	NS	NS
Triglycerides (mmol/L)	2.1 (0.5-12.3)	1.3 (0.4-5.0)	<0.001	0.9 (0.4-2.0)	<0.001	<0.001
Total cholesterol (mmol/L)	5.5 (3.9-11.2)	5.2 (3.2-7.6)	0.012	4.4 (2.2-5.8)	<0.001	<0.001
HDL-C (mmol/L)	1.3 (0.7-2.4)	1.4 (0.6-2.2)	NS	1.4 (0.0-2.3)	NS	NS
LDL-C (mmol/L)	3.1 (1.1-8.2)	3.1 (1.3-4.8)	NS	2.5 (1.4-3.8)	<0.001	<0.001
HbA1c (%)	5.9 (5.0-10.1)	5.6 (4.5-8.6)	<0.001	NA	NA	NA
Insulin (mIU/L)	10.8 (2.61-35.50)	6.43 (0.64-36.3)	<0.001	NA	NA	NA
HOMA-IR	2.88 (0.48-12.37)	1.61 (0.14-10.0)	<0.001	NA	NA	NA
Diabetes (yes/no)	5/80	4/75	NS	NA	NA	NA

Data are reported as median (range) or n (%). *Comparison between MetS OA and Non-MetS OA patients. **Comparison between all OA patients and controls. ***Comparison between Non-MetS OA patients and controls. P-values were calculated by *χ* ^2^ test, *t*-test, or one-way ANOVA, as appropriate. NS: not significant; NA: not applicable. MetS: metabolic syndrome; SBP: systolic blood pressure; DBP: diastolic blood pressure; BMI: body mass index; WHR: waist-to-hip ratio; VAS: visual analogue scale; WOMAC: Western Ontario and McMaster Universities Arthritis Index; HDL-C: high-density lipoprotein-cholesterol; LDL-C: low-density lipoprotein-cholesterol; HbA1c: fasting glycosylated hemoglobin; HOMA-IR: homeostasis model assessment insulin resistance.

Among the patients with OA, blood pressure, BMI, WHR, VAS, and WOMAC were significantly higher in MetS-OA patients compared to those in non-MetS OS patients (P<0.05). Levels of fasting glucose, triglycerides, and total cholesterol were increased in the MetS OA patients compared with non-MetS OA patients (P=0.021, P<0.001, P=0.012, respectively). Similarly, the levels of HbA1c, insulin and HOMA-IR index were significantly higher in patients with MetS OA compared to those with non-MetS OA (P<0.001). Disease duration tended to be higher in MetS OA patients compared to non-MetS OA patients (40.9±50.2 months *vs* 25.3±37.1 months, P=0.025). However, there was no significant difference in HDL-C and LDL-C levels between the two subgroups (P>0.05).

### Levels of leptin and IL-1β

**Figure 1:**
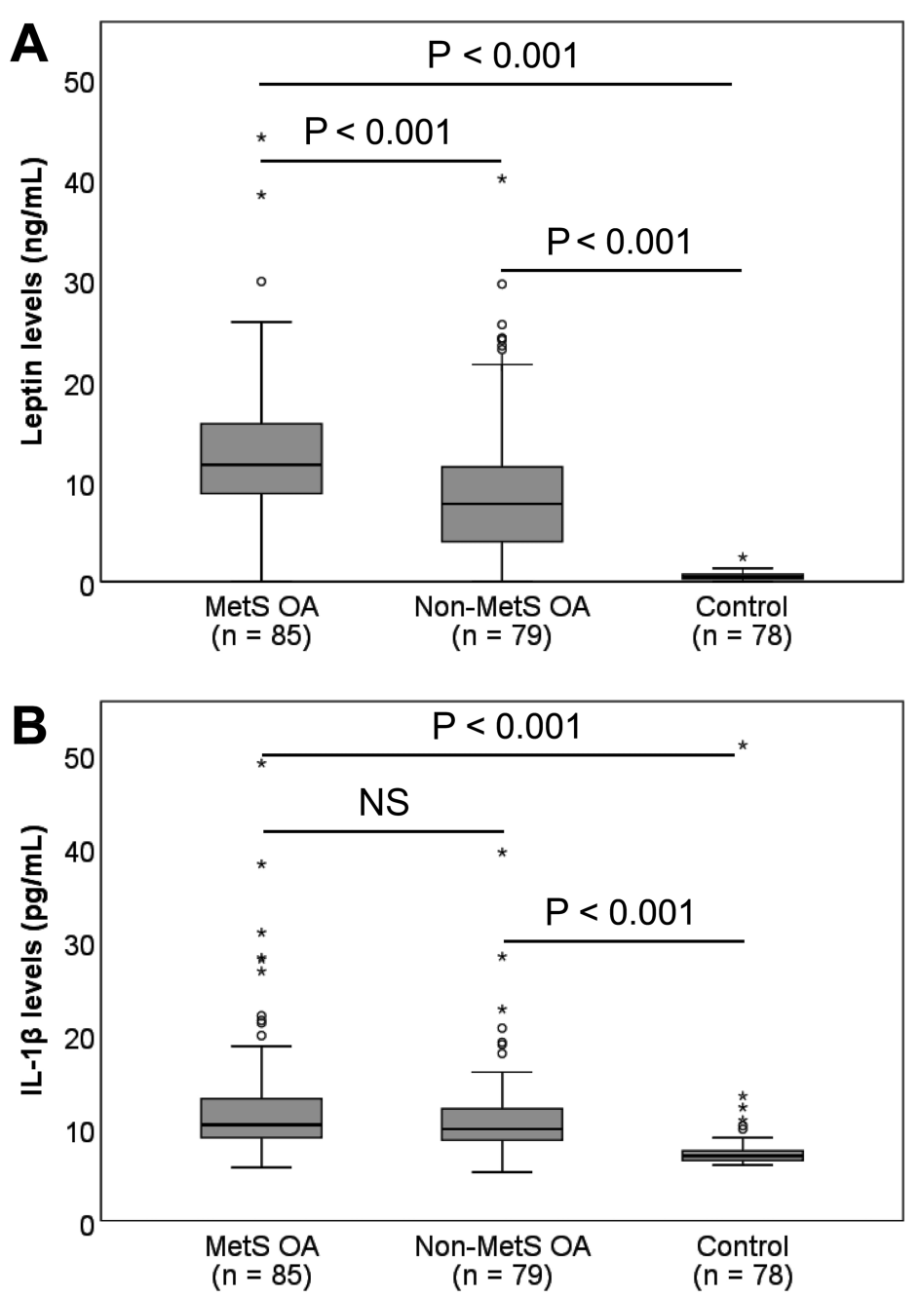
Levels of interleukin-1 beta (IL-1β) and leptin in osteoarthritis (OA) patients and controls. The levels of leptin (**A**) and IL-1β (**B**) were measured in the plasma samples from patients with metabolic syndrome (MetS) OA and non-MetS OA, as well as in control individuals. Data are reported as median and interquartile range. Asterisks indicate outliers. P-values were calculated using the Mann-Whitney U test. NS: not significant.

Patients with OA had significantly higher leptin levels than control individuals (11.50±10.04 *vs* 0.54±0.37 ng/mL, P<0.001). Similarly, IL-1β levels were significantly increased in OA patients compared to controls (14.63±15.87 *vs* 7.79±5.11 pg/mL, P<0.001). Among the OA patients, leptin levels were higher in patients with MetS OA compared to those with non-Met OA (P<0.001). However, no difference in IL-1β levels was observed between the two subgroups (P=0.425). Compared to the control group, both MetS OA and non-MetS OA patients had higher leptin and IL-1β levels (P<0.001) (Figure 1).

**Figure 2:**
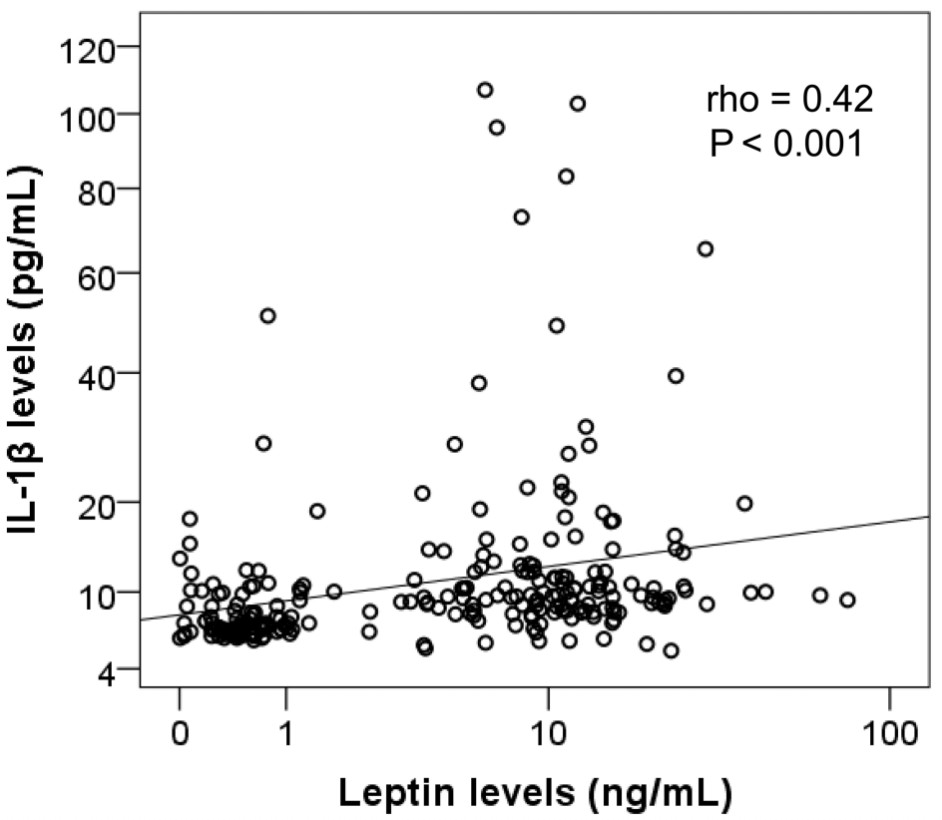
Correlation between interleukin-1 beta (IL-1β) and leptin levels was calculated by using Spearman's rank correlation coefficient.

We conducted further analyses to examine the correlation between IL-1β and leptin levels. The results revealed a significantly positive correlation between IL-1β and leptin levels (Spearman's rho=0.42, P<0.001) (Figure 2). We also compared the levels of leptin and IL-1β among different groups, categorized by age, BMI, number of involved joints, and disease duration. The results showed that leptin levels were significantly elevated with increasing BMI (P<0.001). Moreover, OA patients with two involved joints had higher leptin levels compared to those with only one involved joint (P<0.05). Additionally, patients with a shorter disease duration had higher leptin levels compared to those with a longer disease duration (P<0.05). However, we did not find any significant association between IL-1β levels and these factors, such as BMI, number of involved joints, and disease duration (Table 2).

**Table 2:** Leptin and interleukin-1 beta (IL-1β) levels among different groups categorized by age, body mass index (BMI), number of involved joints, and disease duration in osteoarthritis patients.

Characteristics (n=164)	Leptin (ng/mL)IQR (Q1-Q3)	IL-1β (pg/mL)IQR (Q1-Q3)
Age (years)		
<50	10.6 (6.2-12.0)	10.1 (8.3-12.7)
50-59	8.9 (5.7-13.3)	9.7 (8.5-14.8)
60-69	11.0 (5.7-15.4)	10.1 (9.0-12.5)
≥70	9.2 (2.5-16.4)	10.4 (9.3-14.1)
	NS	NS
Body mass index		
<23	6.2 (3.4-10.3)	10.2 (8.5-13.8)
23-24.9	9.4 (6.9-12.9)	10.1 (8.7-13.0)
≥25	13.0 (10.4-15.8)	9.7 (8.8-12.1)
	P<0.001	NS
Number of involved joints		
1	8.5 (4.0-11.9)	9.6 (8.2-12.2)
2	10.5 (6.3-15.7)	10.3 (9.0-13.2)
	P<0.05	NS
Disease duration (months)		
Short	9.2 (5.4-13.3)	9.2 (8.7-14.0)
Long	11.9 (8.7-15.6)	10.0 (8.9-11.0)
	P<0.05	NS

Data are reported as median and interquartile range (IQR). P-values were calculated by *χ* ^2^ test or Fisher's exact test. NS: not significant.

### Correlation of leptin and IL-1β levels with clinical parameters

The correlation of leptin and IL-1β levels with the clinical parameters of MetS and OA was determined (Table 3). Our findings showed that leptin levels were positively correlated with BMI (Spearman's rho=0.489, P<0.001), WHR (Spearman's rho=0.242, P=0.002), HbA1c (Spearman's rho=0.185, P=0.017), insulin level (Spearman's rho=0.403, P<0.001), HOMA-IR index (Spearman's rho=0.346, P<0.001), and with VAS (Spearman's rho =0.225, P=0.004). IL-1β levels were negatively correlated with insulin levels (Spearman's rho=-0.18, P=0.021) and HOMA-IR index (Spearman's rho=-0.189, P=0.016). A positive correlation between IL-1β and triglyceride levels was also observed, however, it did not reach statistical significance (P=0.05).

**Table 3:** Correlation of leptin and interleukin-1 beta (IL-1β) levels with clinical parameters of metabolic syndrome and osteoarthritis.

Clinical characteristics	Leptin (ng/mL)		IL-1β (pg/mL)	
	(rho)	P-value	(rho)	P-value
Systolic blood pressure (mmHg)	0.036	NS	-0.002	NS
Diastolic blood pressure (mmHg)	-0.019	NS	0.064	NS
Body mass index	**0.489**	**<0.001**	-0.029	NS
Waist-to-hip ratio	**0.242**	**0.002**	0.126	NS
Visual analogue scale	**0.225**	**0.004**	0.086	NS
WOMAC	0.147	NS	-0.061	NS
Fasting glucose (mmol/L)	-0.013	NS	0.070	NS
Triglycerides (mmol/L)	0.014	NS	0.153	0.05
Total cholesterol (mmol/L)	0.136	NS	0.115	NS
HDL-C (mmol/L)	0.087	NS	-0.042	NS
LDL-C (mmol/L)	0.081	NS	0.082	NS
HbA1c (%)	0.185	0.017	-0.110	NS
Insulin (mUI/L)	**0.403**	**<0.001**	0.180	0.021
HOMA-IR	**0.346**	**<0.001**	0.189	0.016

The correlations were calculated using the Spearman's rank correlation coefficient. Bold type indicates statistical significance. NS: not significant. WOMAC: Western Ontario and McMaster Universities Arthritis Index; HDL-C: high-density lipoprotein-cholesterol; LDL-C: low-density lipoprotein-cholesterol; HbA1c: fasting glycosylated hemoglobin; HOMA-IR: homeostasis model assessment insulin resistance.

### Leptin and IL-1β levels as markers of OA and MetS

**Figure 3:**
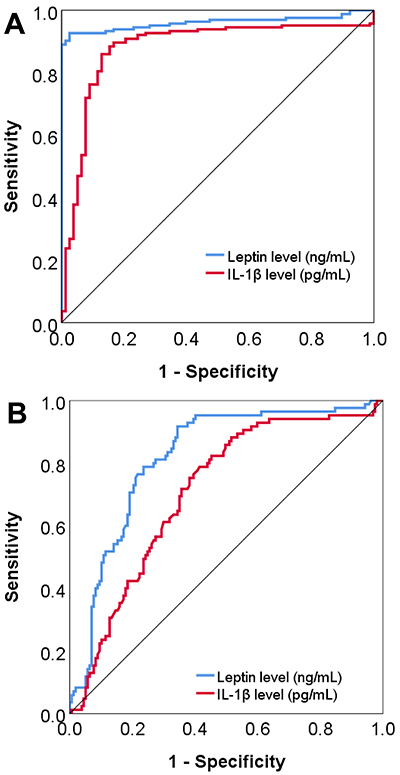
Diagnostic performance of leptin and interleukin-1 beta (IL-1β) levels in distinguishing between individuals with and without osteoarthritis (**A**) and individuals with and without metabolic syndrome (**B**) using ROC curves.

To assess whether leptin and IL-1β levels can distinguish individuals with and without OA, as well as individuals with and without MetS, we conducted a ROC curve analysis. The results demonstrated that both leptin and IL-1β levels had good discriminative power for individuals with and without OA (AUC=0.96 and 0.88, respectively; P<0.001) (Figure 3A). Similarly, the leptin and IL-1β levels could effectively differentiate individuals with and without MetS (AUC=0.82 and 0.71, respectively; P<0.001) (Figure 3B).

## Discussion

Leptin has been implicated in the pathogenesis of obesity, MetS, OA, and inflammation (11,17,20). The findings of this study revealed that plasma leptin levels were significantly increased in patients with OA compared to control individuals. In the OA group, patients with MetS had significantly higher plasma leptin levels than those without MetS. Plasma leptin and IL-1β levels were correlated with several clinical parameters of MetS and OA. These results suggested that leptin and IL-1β could potentially influence the development of both MetS and OA. Notably, leptin and IL-1β may serve as additional markers for distinguishing individuals with MetS and OA from those without MetS and OA.

Research has shown that MetS can influence the pathogenesis and severity of OA (13). In individuals with MetS, metabolic abnormalities such as obesity, insulin resistance, and inflammation can contribute to the development and progression of OA (30). One study reported that the presence of MetS is associated with an increased risk of knee OA progression in a large cohort of patients with knee OA. Specifically, MetS was associated with more severe radiographic knee OA and worsening knee pain and function (10). Similarly, our study also revealed that MetS was associated with increased levels of fasting glucose, triglycerides, total cholesterol, HbA1c, and insulin, and HOMA-IR index in OA patients. Therefore, our results supported the previous finding that the presence of MetS may promote the development and progression of OA by modulating the levels of adipokines and pro-inflammatory cytokines.

Leptin plays an important role in the pathogenesis of MetS via the regulation of metabolism and autoimmune and/or inflammatory processes regarding OA (18,24). A strong relationship between plasma leptin concentration and MetS and OA has been reported (19,31). Our data indicated that plasma leptin levels were significantly higher in patients with OA compared to control individuals and in MetS OA compared to non-MetS OA patients. Plasma leptin levels had a positive correlation with some clinical parameters of MetS, including obesity (BMI and WHR), HbA1c levels, and insulin resistance (insulin level, HOMA-IR index) but were not related to hypertension, hyperglycemia, and dyslipidemia. This result supports the fact that plasma leptin levels were associated with MetS and OA (19), and leptin may be a crucial factor in obese-related OA (17). However, another study concluded that leptin was significantly correlated with BMI, waist circumference, hip circumference, insulin resistance, and lipid parameters (32). The inconsistency between these results may partly be caused by the differences in population and sample size.

We also found that in OA patients, plasma leptin levels were positively correlated with VAS and were higher in patients with more joints involved or longer disease duration. These findings are consistent with other studies that have shown that plasma leptin levels positively correlate with the grade of pain (23), the severity of OA in radiographs, and physical function (21,22). Therefore, we can infer that plasma leptin concentrations have positive associations with VAS and grade of OA. However, Fioravanti et al. (33) reported that plasma levels of leptin were not significantly correlated with the duration of the disease, radiographic severity of knee OA, and VAS. A possible explanation for these results may be the larger sample size and the classification of disease duration. Further studies are needed to confirm the association between leptin and severity of knee OA.

Pro-inflammatory cytokines such as TNF-α, IL-1β, and IL-6, which are mainly produced by immune cells and adipocytes, play a vital role in the pathogenesis of obesity and OA (16). Our observation showed that plasma IL-1β levels in patients with OA were significantly elevated compared to individuals without OA. Patients with OA had increased levels of IL-1β in the synovial fluid, synovial membrane, cartilage, and the subchondral bone layer (34,35), indicating its involvement in the local inflammatory processes that contribute to cartilage degradation and joint destruction. Systemic effects of IL-1β in OA may be particularly relevant in the context of other systemic conditions that are common in OA patients, such as MetS. Although the difference was not statistically significant, we found that IL-1β levels were higher in patients with MetS OA compared to those with non-MetS OA. Therefore, the presence of MetS may contribute to the systemic effects of IL-1β in OA patients. We also found that plasma IL-1β levels were positively correlated with concentrations of circulating leptin, which was established to have an association with OA development (17). This finding is consistent with a previous report showing that leptin induces the production of IL-1β in OA patients (36). Hence, it could be hypothesized that plasma IL-1β importantly contributes to OA pathogenesis. In contrast, no difference in the levels of this marker between the two subgroups of OA was found in our study. Nevertheless, among MetS components, plasma IL-1β levels had a positive correlation with insulin level and HOMA-IR index. These outcomes were in accordance with previous reports that IL-1β can regulate insulin secretion and is associated with insulin resistance (37,38).

Due to the correlation between leptin and MetS as well as OA, plasma leptin levels have been suggested as a predictor of MetS (39), and the ratio of synovial fluid to the plasma leptin level as a marker for the severity of knee OA (21). In this study, ROC curve analyses indicated that plasma leptin levels could serve as an additional marker for differentiating individuals with OA and MetS from those without OA and MetS. IL-1β levels, despite having a lower AUC than leptin levels in predicting both OA and MetS, also showed a good performance in distinguishing individuals with OA. Furthermore, IL-1β concentrations did not correlate significantly with clinical parameters of metabolic syndrome, except for insulin sensitivity/resistance (insulin level and HOMA-IR index).

Although our data indicated a significant association of plasma leptin and IL-1β levels with MetS and OA, this study had several limitations. Firstly, the study design was cross-sectional, and thus, a causal relationship between MetS and OA cannot be determined. The second limitation is that the study focussed on only two markers, whereas MetS and OA are two complex diseases, thus more markers should be studied to clarify the pathogenesis of these two diseases. The third limitation is that the control individuals were younger than the OA patients and we did not have control subgroups (MetS and non-Met controls) like the OA group, which may introduce bias in detecting relationships between the studied markers and MetS, as well as reduce their diagnostic value for MetS.

### Conclusion

Plasma levels of leptin and IL-1β were significantly modulated during the development of MetS and OA, correlated with clinical parameters of MetS and OA, and may play a critical role in the pathogenesis of MetS-related OA. Therefore, leptin and IL-1β levels may serve as additional markers for the diagnosis and prognosis of MetS-related OA. Measuring leptin and IL-1β levels in plasma may be useful in identifying individuals who could benefit from interventions aimed at modulating leptin and IL-1β signaling in OA.
